# Cardiovascular magnetic resonance detects the progression of impaired myocardial perfusion reserve and increased left-ventricular mass in mice fed a high-fat diet

**DOI:** 10.1186/s12968-016-0273-y

**Published:** 2016-09-09

**Authors:** Nivedita K. Naresh, Joshua T. Butcher, Robert J. Lye, Xiao Chen, Brant E. Isakson, Li-Ming Gan, Christopher M. Kramer, Brian H. Annex, Frederick H. Epstein

**Affiliations:** 1 Department of Biomedical Engineering, University of Virginia, Charlottesville, VA USA; 2 Robert M. Berne Cardiovascular Research Center, University of Virginia, Charlottesville, VA USA; 3 Department of Molecular Physiology and Biological Physics, University of Virginia, Charlottesville, VA USA; 4 Department of Molecular and Clinical Medicine, AstraZeneca R&D, Mölndal, Sweden; 5 Institute of Medicine, Sahlgrenska Academy, CVMD Early Clinical Development, AstraZeneca R&D, Mölndal, Sweden; 6 Cardiovascular Medicine, University of Virginia, Charlottesville, VA USA; 7 Department of Radiology, University of Virginia, Charlottesville, VA USA

**Keywords:** Myocardial perfusion reserve, Mouse, Cardiovascular magnetic resonance, Obesity, Type 2 diabetes mellitus

## Abstract

**Background:**

Impaired myocardial perfusion reserve (MPR) is prevalent in obesity and diabetes, even in the absence of obstructive coronary artery disease (CAD), and is prognostic of adverse events. We sought to establish the time course of reduced MPR and to investigate associated vascular and tissue properties in mice fed a high-fat diet (HFD), as they are an emerging model of human obesity, diabetes, and reduced MPR without obstructive CAD.

**Methods:**

C57Bl/6 mice fed a HFD or a low-fat diet (control) were imaged at 6, 12, 18 and 24 weeks post-diet. The cardiovascular magnetic resonance (CMR) protocol included multi-slice cine imaging to assess ejection fraction (EF), left-ventricular (LV) mass, LV wall thickness (LVWT), and LV volumes, and first-pass perfusion CMR to quantify MPR. Coronary vascular reactivity, aortic atherosclerosis, myocardial capillary density and tissue fibrosis were also assessed.

**Results:**

Body weight was increased in HFD mice at 6–24 weeks post-diet (*p* < 0.05 vs. control). MPR in HFD mice was reduced and LV mass and LVWT were increased in HFD mice at 18 and 24 weeks post-diet (*p* < 0.05 vs. control). Coronary arteriolar vascular reactivity to adenosine and acetylcholine were reduced in HFD mice (*p* < 0.05 vs. control). There were no significant differences in cardiac volumes, EF, or capillary density measurements between the two groups. Histology showed interstitial fibrosis in HFD and no aortic atherosclerosis in either group.

**Conclusions:**

C57Bl/6 mice fed a HFD for 18–24 weeks have progressively increased LV mass and impaired MPR with fibrosis, normal capillary density and no aortic plaque. These results establish C57Bl/6 mice fed a HFD for 18–24 weeks as a model of impaired MPR without obstructive CAD due to obesity and diabetes.

## Background

For several decades the prevailing practice in the treatment of ischemic heart disease has equated myocardial ischemia with obstructive coronary artery disease (CAD) [1]. Accordingly, most therapies for myocardial ischemia aim to remove coronary stenoses. However, there is mounting evidence that not all patients with ischemia have obstructive CAD, and a paradigm shift has been proposed [1]. The emerging concept is that multiple factors including microvascular disease, coronary vasospasm, and others may be significant contributors to myocardial ischemia, and that obstructive CAD is one of multiple potential causes [1]. Indeed, studies have shown that myocardial ischemia in the absence of obstructive CAD occurs with considerable prevalence in certain patient populations including diabetics [2], obesity [3], the metabolic syndrome [4] and women [5].

The presence and severity of myocardial ischemia can be assessed quantitatively by imaging myocardial perfusion reserve (MPR) using PET or cardiovascular magnetic resonance (CMR). Reduced MPR has been shown to be prognostic of adverse cardiovascular events and an independent predictor of cardiac mortality in patients with and without obstructive CAD [6]. One recent study with more than 1000 diabetic and 1600 non-diabetic subjects showed that diabetic patients without obstructive CAD but with impaired MPR have the same event rate as non-diabetic patients with prior CAD [2]. In the absence of obstructive CAD, impaired MPR largely reflects dysfunction of the resistance vessels of the microvasculature. While microvascular dysfunction leads to reduced MPR and increased cardiovascular risk, the mechanisms underlying microvascular dysfunction are not completely understood, and corresponding treatment strategies are not established.

Mouse models are widely utilized in cardiovascular research to study underlying molecular mechanisms, and mice fed a high-fat diet (HFD) are emerging as a common model of human obesity and diabetes. A recent study characterized the long term effects of a HFD on the cardiovascular system in mice and found that mice fed a HFD for 8–16 months develop obesity, hyperglycemia, hyperinsulinemia, insulin resistance, cardiomyocyte hypertrophy and cardiac metabolic maladaptations [7]. Another recent study used CMR to show that HFD mice develop diastolic dysfunction after 20 weeks of diet [8]. In addition, we recently used first-pass contrast-enhanced perfusion CMR to demonstrate that HFD mice have impaired MPR at 24 weeks after initiating the diet [9]. In the present study we sought to establish the time course and investigate vascular and tissue properties underlying the progression of increased LV mass and reduced MPR in HFD mice.

## Methods

### Experimental design

Two groups of mice were studied: C57Bl/6 mice fed a HFD (*n* = 11) (60 % calories from fat, Diet 12492, Research Diets Inc., New Brunswick, NJ) and age-matched C57Bl/6 control mice fed a low-fat diet (*n* = 9) (10 % calories from fat, Diet D12450J, Research Diets Inc.). Male mice were selected for this study as prior studies have shown that female C57Bl/6 mice are protected against HFD-induced glucose intolerance [10] and the development of obesity-related cardiac dysfunction [11]. Over the course of this study, three HFD mice and one control mouse died, bringing the sample size to *n* = 8 in each group by the end of the study. Mice were started on their respective diets at 6 weeks of age, and were studied at 6, 12, 18 and 24 weeks post-diet. The CMR protocol at all time points included (a) perfusion imaging at rest and with a vasodilator, (b) cine displacement encoding with stimulated echoes (DENSE) imaging to measure myocardial strain, and (c) cine CMR to measure end-diastolic volume (EDV), end-systolic volume (ESV), ejection fraction (EF), LV mass, LV wall thickness at end-diastole (LV EDWT) and LV wall thickness at end-systole (LV ESWT). Glucose tolerance tests (GTTs) were performed at 6, 12, 18 and 24 weeks post-diet. Systolic blood pressure was measured in conscious mice at 25 weeks post-diet using a non-invasive tail cuff plethysmography system (Model BP 2000, Visitech Systems, Apex, NC). Mice were euthanized at 26 weeks post-diet for histology and to assess coronary vascular reactivity.

### Animal handling

All animal studies were performed under protocols that comply with the Guide for the Care and Use of Laboratory Animals (NIH publication no. 85–23, Revised 1996) and were approved by the Animal Care and Use Committee at our institution. An indwelling tail vein catheter was established to deliver Gd-DTPA (Magnevist, 0.1 mM/kg body weight) and Regadenoson (Lexiscan, Astellas Pharmis, 0.1 μg/g body weight) during CMR. Body temperature was maintained at 36 ± 0.5 °C and anesthesia was maintained using 1.1–1.25 % isoflurane in O_2_. Body weight was recorded for all animals before starting the diets and at the beginning of each imaging study.

### Glucose tolerance tests

For GTTs [12], mice were injected intraperitoneally with 1 g/kg glucose in milli-Q water after overnight fasting for 15–16 hours. A tail vein blood sample was taken before injection of glucose to measure the fasting blood glucose and at 10, 30, 60 and 90 min post-injection of the glucose solution. The area under the curve (AUC) was calculated to evaluate glucose tolerance using the trapezoidal rule [7].

### CMR acquisitions

CMR was performed on a 7T Clinscan system (Bruker, Ettlingen, Germany) using a 30–35 mm diameter birdcage RF coil. The bigger coil was used when necessary to accommodate the heavier mice. Localizer imaging was performed to select a mid-ventricular short-axis slice. Rest perfusion was then imaged using a compressed sensing (CS)-accelerated dual-contrast first-pass sequence [9]. Using this sequence, two slices were acquired: one to sample the arterial input function (AIF) and the other to sample the tissue function (TF). Imaging parameters included: echo time/repetition time (TE/TR) = 1.2/2.1 ms, field of view (FOV) = 25.6 × 18 mm^2^, matrix = 128 × 74, phase FOV = 72 %, percent sampling = 80 %, image resolution = 200 × 250 μm^2^, flip angle = 15^0^, slice thickness = 1 mm, AIF saturation delay = 15 ms, TF saturation delay = 57 ms, AIF acceleration rate = 6, TF acceleration rate = 4, AIF acquisition time = 25 ms/image and TF acquisition time = 36 ms/image. Thereafter, baseline LV structure and function were assessed using a black-blood cine CMR sequence as described previously [13]. Six - eight short-axis slices were acquired covering the entire LV from base to apex. Imaging parameters included: TE/TR = 1.9/4.4 ms, temporal resolution = 4.4 ms, slice thickness = 1 mm, and image resolution = 200 μm^2^. Myocardial strain was then measured in a mid-ventricular short-axis slice using the cine-DENSE method as previously described [14, 15]. For the cine DENSE acquisition, fat-saturation was applied when necessary to null the signal from fat in order to improve the image quality. Thereafter, Regadenoson was injected i.v. and 10 min later first-pass CMR was repeated.

### Vascular reactivity

After 26 weeks on diet, a subgroup of HFD and control mice were euthanized and coronary arteries (*n* ≥ 4) from the second arborized branches off the left coronary artery were isolated (lumen diameter = 90.9 ± 9.5 μm). The arteries were freed of the surrounding cardiac myocytes and were placed in an arteriograph (Danish MyoTechnology, DMT, Ann Harbor, MI), where they were cannulated at both ends and pressurized to 40 mmHg as previously described [16–18]. Cumulative dose-responses to adenosine and acetylcholine and a step-wise pressure increase for assessment of passive tone were measured as previously described [16–18].

### Histology

Mice were euthanized for histology (*n* = 5, each group), hearts were harvested and mid-ventricular short-axis sections (3 mm thick) were fixed in 4 % paraformaldehyde for 4 hours and then embedded in paraffin. Sections (5 μm thick) were stained with anti-CD31 antibody (Santa Cruz Biotechnology) to quantify capillary density, and Masson’s Trichrome to quantify interstitial fibrosis and perivascular fibrosis. For the assessment of systemic atherosclerosis, ascending aortas were excised and stained with Sudan IV.

For histology, image analysis was performed using ImageJv1.49 g (NIH). Quantification of capillary density was performed on 6 random fields (40x) and capillaries and cardiomyocytes were counted using the ‘analyze particles’ function in ImageJ. Quantification of interstitial fibrosis was performed on 15 random fields (40x) (excluding the blood vessels) and a thresholding method was used to quantify the area occupied by collagen as a percentage [19]. Quantification of perivascular fibrosis was performed on 2 arteries per mouse (40x) and the average perivascular collagen area normalized to the vessel luminal area was recorded. The aortic plaque area, quantified using ImageJ, was expressed as a percentage of the aortic vessel area.

### Analysis of MR images

For perfusion images, image reconstruction and analysis were performed in MATLAB (Mathworks, Natick, MA) as previously described [9]. Undersampled first-pass perfusion images were reconstructed using Block LOw-rank Sparsity with Motion-guidance (BLOSM), a motion-compensated CS method [20]. Perfusion analysis was based on Fermi function deconvolution [21]. Briefly, signal intensity vs. time curves were obtained by placing a region of interest (ROI) in the LV blood pool for the AIF and in the myocardium for the TF. These signal intensities were normalized by the signal intensities of proton-density weighted images and thereafter were converted into T1 values using the methods described by Cernicanu and Axel [22]. The pre-contrast T1 was fixed at 1.55 s for blood and 1.45 s for myocardium [9]. Using Fermi function deconvolution, rest and stress perfusion were quantified for each mouse at each time point. MPR for each mouse at each time point was then calculated as the ratio of stress perfusion to rest perfusion. Some mice were excluded for perfusion studies due to difficulty in repetitive placement of i.v. lines and due to bad image quality of stress perfusion images (caused due to motion artifacts at high heart-rates). The sample sizes at each time point were as follows: *n* = 7 (HFD) and *n* = 6 (Control) at 6 weeks post-diet, *n* = 6 (HFD) and *n* = 6 (Control) at 12 weeks post-diet, *n* = 8 (HFD) and *n* = 9 (Control) at 18 weeks post-diet, *n* = 8 (HFD) and *n* = 7 (Control) at 24 weeks post-diet. The black-blood cine images were imported to a workstation and analyzed using Segment software (Medviso, AB). Specifically, the end-diastolic (ED) and end-systolic (ES) frames were identified and thereafter the endocardial and epicardial contours were drawn on these frames for all the slices. Using the software, the EDV, ESV, EF, LV mass, LV EDWT and LV ESWT were calculated. Strain analysis of cine DENSE images was performed using the DENSE analysis tool [23–25], which is a semi-automatic technique implemented in MATLAB. Global peak circumferential strain (E_cc_) was used as another metric of systolic function. As done previously for HFD mice [26], subepicardial circumferential strain (E_cc-subepi_) and subendocardial circumferential strain (E_cc-subendo_) were also assessed. Cine DENSE strain data were also used to quantify LV synchrony using the circumferential uniformity ratio estimate (CURE) [27, 28].

### Statistical analysis

Statistical analysis was performed using SigmaPlot (Systat Software Inc., Point Richmond, CA). Differences in body weight, EDV, ESV, EF, LV mass, LV EDWT, LV ESWT, rest perfusion, stress perfusion, MPR, global E_cc_, E_cc-subepi_, E_cc-subendo_ and CURE were analyzed using two-way repeated measures analysis of variance (ANOVA). All values in text, tables and graphs are presented as mean ± standard deviation.

## Results

### Body mass and glucose tolerance tests

**Fig. 1:**
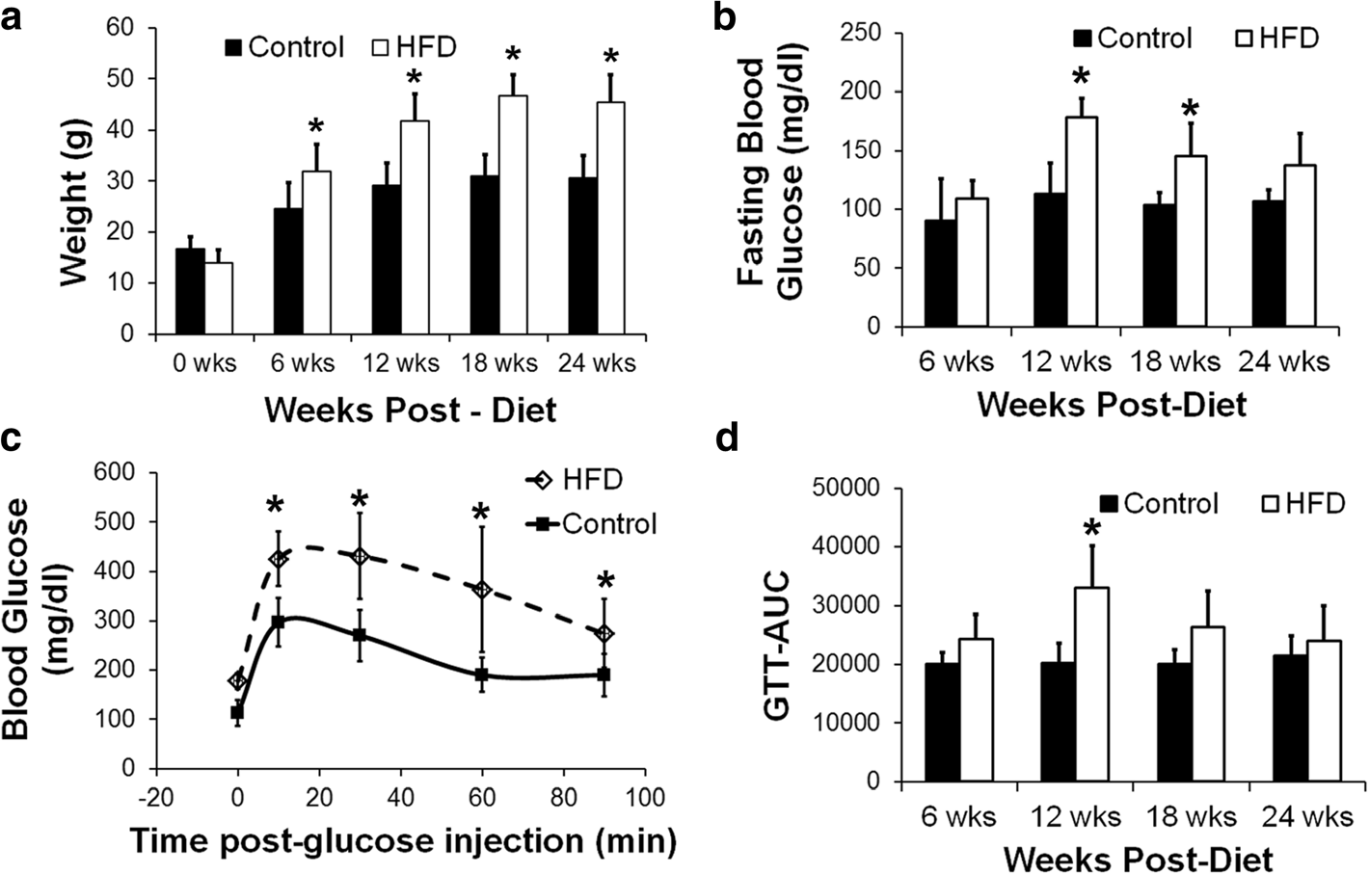
**a** Body weight measurements in mice fed high-fat diet (HFD) and low-fat diet (control) at the start of diet and 6, 12, 18 and 24 weeks post-diet (*p < 0.05 vs. age-matched control). **b** Fasting blood glucose measurements in control and HFD mice at 6, 12, 18 and 24 weeks post-diet (*p < 0.05 vs. age-matched control). **c** Glucose tolerance curves in the control and HFD mice at 12 weeks post-diet (*p < 0.05 vs control at the same time-point). **d** The area under the curve (AUC) of the glucose tolerance test at 6, 12, 18 and 24 weeks post-diet (*p < 0.05 vs. age-matched control)

Body weight was significantly higher in the HFD mice at 6 weeks post-diet and it progressively increased with time (Fig. 1a, *p* < 0.05 vs. age-matched control). The HFD mice were hyperglycemic at 12 and 18 weeks post-diet as measured using fasting blood glucose (Fig. 1b, *p* < 0.05 vs. age-matched control). The HFD mice were glucose intolerant as compared to the control mice at 12 weeks post-diet (Fig. 1c–d, *p* < 0.05 vs. age-matched control).

### CMR

#### Perfusion

**Fig. 2:**
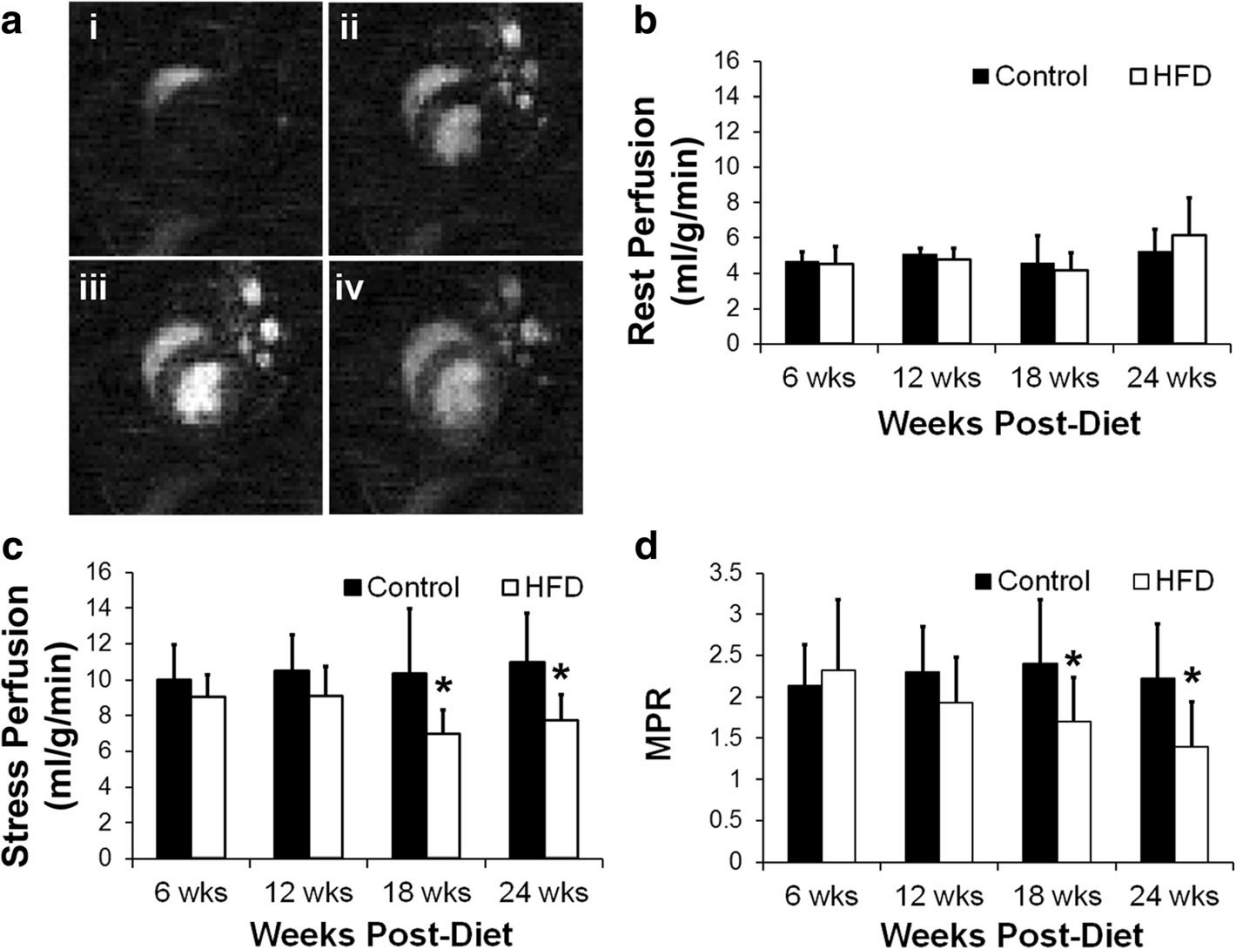
**a** Example first-pass perfusion images obtained from a mouse at rest. These images show the arrival of contrast agent in the right ventricle (i), left ventricle (ii and iii) and the myocardium (iv). **b** Rest perfusion measurements in the control and HFD mice at 6, 12, 18 and 24 weeks post-diet. **c** Stress perfusion measurements in the control and HFD mice at 6, 12, 18 and 24 weeks post-diet (*p < 0.05 vs. age-matched control). **d** Myocardial perfusion reserve (MPR) measurements in the control and HFD mice (*p < 0.05 vs. age-matched control)

Figure 2a shows example first-pass perfusion images obtained from a mouse heart. There were no differences in the rest perfusion measurements between the two groups of mice at any of the time points (Fig. 2b). However stress perfusion in the HFD mice was reduced as compared to control mice at 18 and 24 weeks post-diet (Fig. 2c, *p* < 0.05 vs. age-matched control). MPR was also reduced in the HFD mice as compared to control mice at 18 and 24 weeks post-diet (Fig. 2d, *p* < 0.05 vs. age-matched control).

#### LV structure and function

**Fig. 3:**
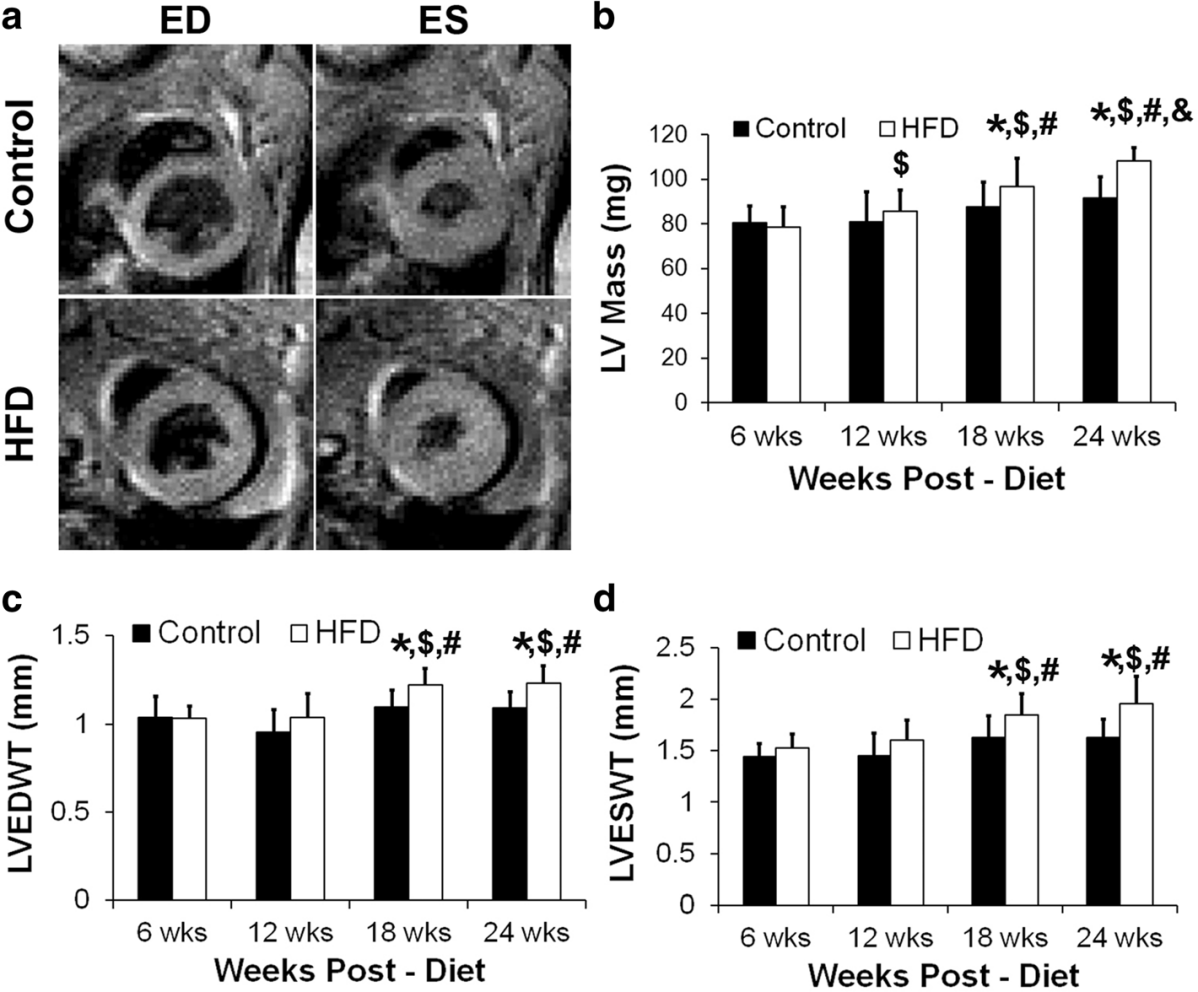
**a** Example black-blood cine images in the control and HFD mice at end-diastole and end-systole at 18 weeks post-diet. **b** LV mass measurements in the control and HFD mice at 6–24 weeks post-diet (*p < 0.05 vs. age-matched control, $*p* < 0.05 vs. HFD at 6 weeks, #*p* < 0.05 vs. HFD at 12 weeks, &*p* < 0.05 vs. HFD at 18 weeks). **c** LV end-diastolic wall thickness (LVEDWT) measurements in the control and HFD mice at 6–24 weeks post-diet (*p < 0.05 vs. age-matched control, $*p* < 0.05 vs. HFD at 6 weeks, #*p* < 0.05 vs. HFD at 12 weeks). **d** LV end-systolic wall thickness (LVESWT) measurements in the control and HFD mice at 6–24 weeks post-diet (*p < 0.05 vs. age-matched control, $*p* < 0.05 vs. HFD at 6 weeks, #*p* < 0.05 vs. HFD at 12 weeks)

Figure 3a shows example black-blood cine images obtained from a control and a HFD mouse at ED and ES at 18 weeks post-diet. LV mass was significantly higher in the HFD mice as compared to the control mice at 18 and 24 weeks post-diet (Fig. 3b, *p* < 0.05 vs. age-matched control). LV mass progressively increased in the HFD mice from the start of the diet to the end of the study. We also saw a slight increase in LV mass in the control group at 24 weeks post-diet (*p* < 0.05 vs. control at 6 and 12 weeks post-diet), probably due to normal growth at their age. LV EDWT and ESWT were also significantly increased in the HFD mice at 18 and 24 weeks post-diet (Fig. 3c–d, *p* < 0.05 vs. age-matched control, *p* < 0.05 vs. HFD at 6 and 12 weeks post-diet). There were no statistically significant differences in LV EDV, LV ESV, LV EF, LV E_cc-subendo_, LV E_cc-subepi_, LV global E_cc_ or CURE measurements between the two groups of mice over the time course (Table 1).

**Table 1:** CMR parameters of cardiac function

	Time (Weeks after start of diet)							
	6 weeks		12 weeks		18 weeks		24 weeks	
	Control	HFD	Control	HFD	Control	HFD	Control	HFD
LVEDV (μL)	43 ± 7	42 ± 7	48 ± 9	47 ± 10	44 ± 7	40 ± 7	40 ± 6	43 ± 5
LVESV (μL)	17 ± 4	14 ± 4	17 ± 6	17 ± 6	16 ± 6	12 ± 5	13 ± 4	11 ± 5
LVEF (%)	61 ± 8	66 ± 8	64 ± 12	65 ± 8	64 ± 9	69 ± 12	67 ± 8	74 ± 11
Heart Rate (bpm)	530 ± 63	538 ± 88	478 ± 39	485 ± 69	450 ± 84	512 ± 62	507 ± 47	549 ± 59
Change in Heart Rate under Stress (%)	23 ± 16	28 ± 12	27 ± 5	16 ± 13	15 ± 19	21 ± 13	21 ± 28	−2 ± 15*
E_cc_	−0.14 ± 0.02	−0.13 ± 0.02	−0.13 ± 0.02	−0.12 ± 0.01	−0.12 ± 0.03	−0.11 ± 0.02	−0.12 ± 0.01	−0.14 ± 0.02
E_cc-subepi_	−0.09 ± 0.01	−0.09 ± 0.02	−0.09 ± 0.01	−0.08 ± 0.01	−0.08 ± 0.02	−0.07 ± 0.02	−0.08 ± 0.01	−0.09 ± 0.01
E_cc-subendo_	−0.19 ± 0.02	−0.17 ± 0.03	−0.18 ± 0.03	−0.16 ± 0.02	−0.17 ± 0.04	−0.16 ± 0.03	−0.17 ± 0.02	−0.20 ± 0.03
CURE	0.87 ± 0.06	0.85 ± 0.07	0.90 ± 0.05	0.87 ± 0.05	0.85 ± 0.06	0.89 ± 0.04	0.84 ± 0.04	0.84 ± 0.03

*p < 0.05 vs. Control at same time point

### Blood pressure and heart rate

Systolic blood pressure at 25 weeks post-diet was found to be 106 ± 7 mmHg in the control group and 110 ± 7 mmHg in the HFD group. Diastolic blood pressure at 25 weeks post-diet was found to be 81 ± 8 mmHg in the control group and 80 ± 9 mmHg in the HFD group. These differences were not statistically significant. There were also no significant heart rate differences between the two groups of mice for the entire time course of the study (Table 1).

### Vascular reactivity

**Fig. 4:**
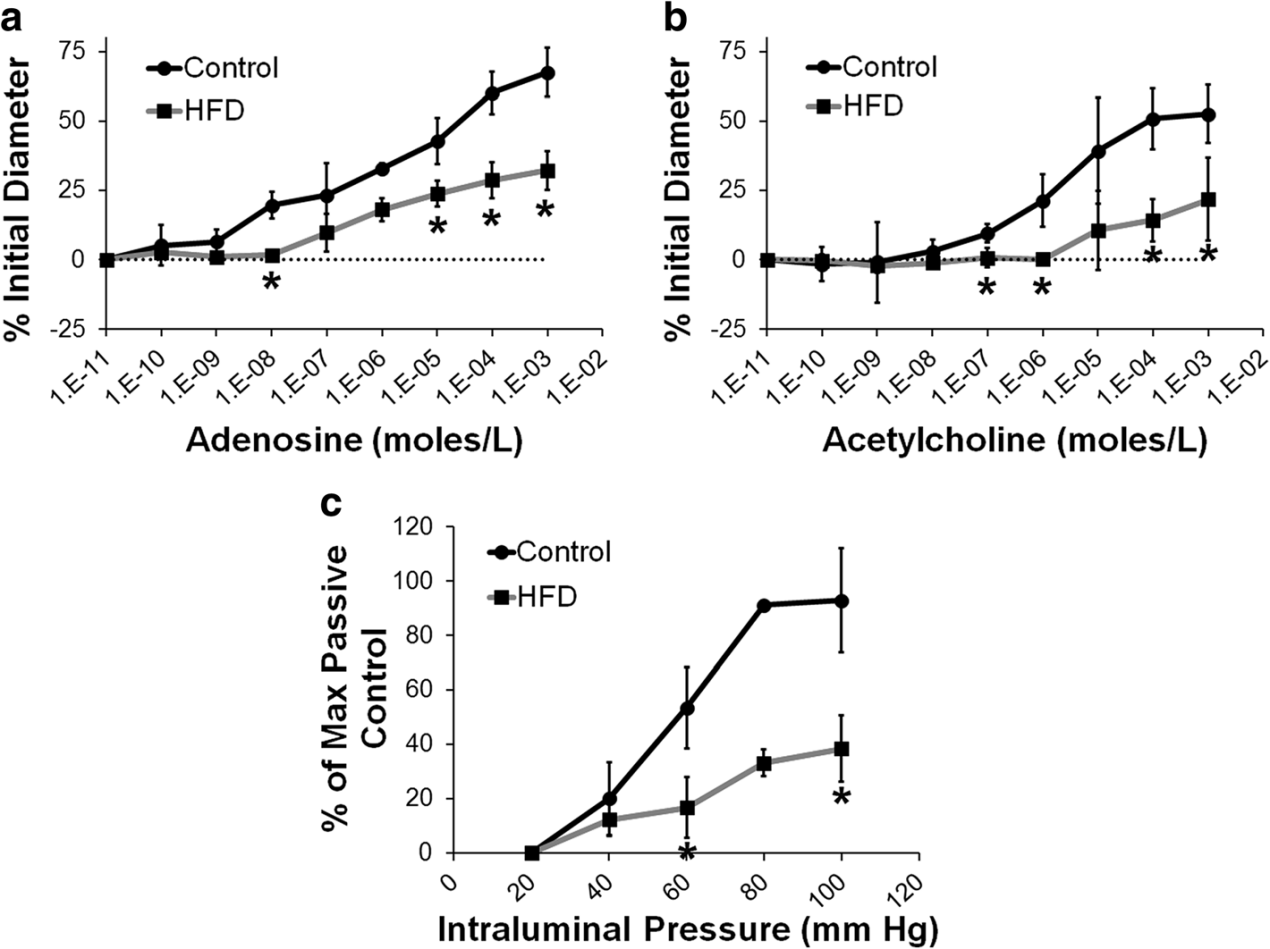
**a** Cumulative arteriolar dose-response curves to adenosine in control and HFD mice at 26 weeks post-diet (*p < 0.05 vs control at same concentration). **b** Cumulative dose-response curves to acetylcholine in control and HFD mice at 26 weeks post-diet (*p < 0.05 vs. control at same concentration). **c** Passive tone in the control and HFD mice at 26 weeks post-diet (*p < 0.05 vs. control at same pressure)

Cumulative dose-response curves to adenosine demonstrated a significantly decreased ability of the arterioles to dilate in mice fed a HFD for 26 weeks (Fig. 4a, *p* < 0.05 vs. control). Since loss of endothelial-dependent dilation is a hallmark of HFD, we tested vascular reactivity to acetylcholine, an endothelial specific dilator. Similar to adenosine, the ability of HFD mice to dilate coronary arterioles was significantly inhibited in response to acetylcholine (Fig. 4b, *p* < 0.05 vs. control). Lastly, we tested whether matrix composition may also be altered in the coronary arterioles by measuring the passive tone in response to increasing pressure steps. In the HFD mice, the passive tone was significantly inhibited as compared to the control mice (Fig. 4c, *p* < 0.05 vs. control). These results demonstrated that the coronary arterioles in the HFD mice have a significantly decreased capacity to dilate.

### Histology

**Fig. 5:**
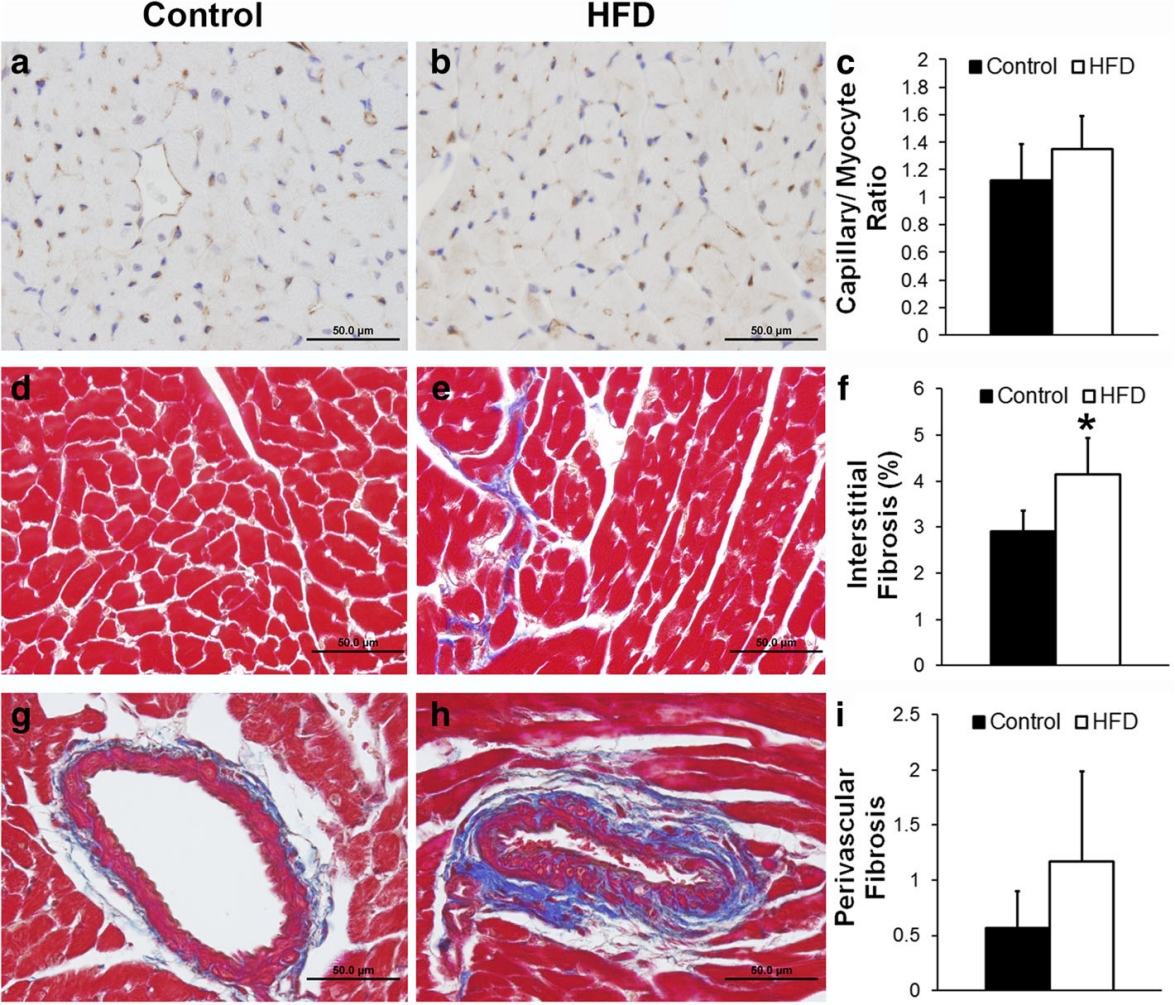
**a** Representative CD31-stained sections of the heart from a control and (**b**) a HFD mouse. **c** Capillary density in control and HFD mice. **d** Representative Masson’s Trichrome stained sections of the heart from a control and (**e**) a HFD mouse. **f** Myocardial interstitial fibrosis in control and HFD mice (*p < 0.05 vs. control). **g** Representative Masson’s Trichrome-stained coronary vessels from sections of the heart from a control mouse and (**h**) a HFD mouse. **i** Perivascular fibrosis in control and HFD mice. Scale bars = 50 μm

Figure 5 shows example CD31-stained images from a control (Fig. 5a) and a HFD mouse heart (Fig. 5b). There were no significant differences in the number of capillaries per cardiomyocyte between the two groups of mice (Fig. 5c). Furthermore, there was no aortic atherosclerosis in either control or HFD mice. Aortic plaque was found to be 3.7 ± 1.8 % in the control mice and 3.2 ± 1.4 % in the HFD mice. Figure 5 shows Masson’s Trichrome stained sections of myocardium obtained from a control mouse (Fig. 5d) and a HFD mouse (Fig. 5e). We found increased interstitial fibrosis in HFD mice (Fig. 5f, *p* < 0.05 vs. control) at 26 weeks post-diet. Figure 5 also shows Masson Trichrome-stained coronary vessels obtained from a control mouse (Fig. 5g) and a HFD mouse (Fig. 5h). We found a trend towards increased perivascular fibrosis in HFD mice (Fig. 5i).

## Discussion

In this study we established the time course of impaired MPR in mice fed a HFD and related the impaired MPR to underlying dysfunction of the coronary arterioles. Using serial in vivo noninvasive CMR we showed progressively reduced MPR at 18 and 24 weeks of HFD in C57Bl/6 mice without changes in capillary density and without aortic atherosclerosis. It is established that C57Bl/6 mice without additional genetic manipulation do not develop obstructive atherosclerosis in the aorta or coronary arteries [29], and our measurements in the aorta are consistent with these prior findings. The MPR imaging results were supported by ex vivo vascular reactivity studies, which showed an impaired vasodilatory response of the coronary arterioles of HFD mice to adenosine. Coronary arterioles from HFD mice also had a reduced response to acetylcholine, showing endothelial dysfunction as well as impaired passive tone in response to increasing pressure steps. The finding that impaired MPR occurs after 18 weeks of HFD is consistent with the recently-established time course for impaired systolic strain with dobutamine stress, which occurred at 22 weeks of HFD but not 16 weeks [30]. Furthermore, we showed that 18 weeks of HFD in mice resulted in increased LV mass and increased LVWT without changes in LV volumes and peak LV E_cc_. Our results in this mouse model are consistent with prior studies in patients with obesity and diabetes which have shown that MPR is progressively reduced in patients with increasing body weight [3] and in type-2 diabetic patients with increasing haemoglobin A1c [31].

Our vascular reactivity results are consistent with prior studies by Yamamoto et al. [32] and Calligaris et al. [7], who have shown impaired endothelium-dependent relaxation in excised thoracic aortas in response to acetylcholine in HFD mice at 17 weeks and at 8–16 months post-diet. Furthermore, these results are in line with prior studies which reported impaired coronary endothelial function in obese patients [3, 33].

In this study, we showed that HFD mice develop increased LV mass and LVWT after 18 weeks of feeding. However, there were no significant differences in LV systolic function (EF, E_cc_, synchrony) between the two groups of mice. These results agree with a recent study by Calligaris et al. [7] who showed increased LV mass, cardiomyocyte hypertrophy and normal systolic function in HFD mice at 8–16 months of HFD feeding (60 % calories from fat) using histology, echocardiography and cardiac catheterization. Our results also agree with a recent cine DENSE study of HFD mice [30], but disagree with an older study [26]. In the older study [26], mice fed a HFD for 5 months showed impaired systolic strain, torsion and synchrony. However, more recent results from this group [30] showed that dobutamine-induced systolic strain impairments occur in mice fed a HFD for 22 weeks, however impaired systolic strain at rest is not observed until 42 weeks of diet. Another recent study using high frame-rate cine CMR also showed normal systolic function in HFD mice (45 % calories from fat) at 20 weeks post-diet and, importantly, demonstrated diastolic dysfunction [8]. Synthesizing results from multiple studies, the pathophysiology time course appears to include impaired MPR and increased LV mass and wall thickness by 18 weeks of HFD, diffuse and perivascular fibrosis, dobutamine-inducible impairments in strain, and possibly subtle diastolic dysfunction around weeks 20–25 of HFD, and impaired resting strain by 42 weeks of HFD.

Previous studies have used C57Bl/6 mice with different specific diet compositions to study the effects of obesity, diabetes and metabolic syndrome on cardiac function [7, 8, 11, 32, 34–37]. Diets vary in terms of percentage of calories from fat (30–78 %), and in the amounts of carbohydrate, protein, fatty acids, and sugar [38]. The specific diet composition can alter the degree of weight gain and the metabolic pathophysiology. In part due to the variety of diets, prior studies investigating the effect of HFD on cardiac function have yielded conflicting results [8, 26, 34].

The mechanisms underlying impaired MPR and coronary vascular reactivity are not completely understood. Deterioration of the overall endothelium or endothelial dilatory components is a possibility. Changes in eNOS have been observed in several HFD models [39–41]. Impaired responses to both adenosine and acetylcholine, agonists that have well-defined endothelial derived nitric oxide release for smooth muscle dilation, would indicate this is likely the case. Furthermore, we also found that the passive tone in isolated coronary arterioles was significantly reduced, suggesting a change in the extracellular matrix composition, making the arterioles more rigid. Correspondingly, using histology, we also showed significantly higher interstitial fibrosis and a trend towards increased perivascular fibrosis in the HFD mice. Similarly, a prior study showed that mice fed a HFD develop increased perivascular fibrosis [32]. This change in extracellular matrix, especially perivascular fibrosis, may result in stiffer vessels, thereby affecting the dilatory capacity of these vessels. Regardless of the exact mechanism underlying the change in vascular reactivity, because the ability of arterioles to dilate or constrict governs the degree of blood flow to the capillaries, it is highly likely that the inability of these coronary arterioles to dilate in response to adenosine (or acetylcholine and changes in pressure) is responsible for the significantly reduced MPR detected by noninvasive imaging.

Several mechanisms have been implicated in the pathogenesis of obesity- [42] and diabetes [43]-related cardiac hypertrophy such as insulin resistance, lipotoxicity [44, 45], inflammation [46], oxidative stress and renin-angiotensin-aldosterone system (RAAS) activity. Recent studies suggest that low levels of adiponectin in obesity and diabetes-related disorders may also contribute to cardiac hypertrophy [47, 48]. Some or all of the above mentioned mechanisms may be responsible for the increased LV mass observed in the HFD mice in this study. With regard to impaired MPR, a prior study showed that Interleukin-6 (IL-6), a cytokine associated with obesity, can induce oxidative stress and endothelial dysfunction by upregulating the angiotensin II type 1 receptor [49]. Additionally, a recent study showed that Olmesartan, an angiotensin II type 1 receptor blocker, significantly suppressed HFD-induced vascular endothelial dysfunction and disruption of eNOS in the thoracic aorta in mice [32]. Thus, upregulation of the angiotensin II type 1 receptor may be responsible for the reduction in MPR observed in the HFD mice in this study. Several factors such as myocardial lipid accumulation and lipotoxicity may contribute to the increased cardiac fibrosis observed in the mice fed a high-fat diet. A recent study by Abdesselam et al. [50] showed increased myocardial lipid accumulation along with increased cardiac fibrosis in mice fed a high-fat and high-sucrose diet. Furthermore, Glenn et al. [44] showed increased cardiac fibrosis in a mouse model of cardiac steatosis.

Over the time course of the present study, three HFD mice and one control mouse died. The mice died in between the imaging sessions, not during CMR or glucose tolerance testing. One of the high-fat diet mice that died developed an abdominal tumor and the other two high-fat diet mice had small abdominal lesions. Including the present study as well as other studies in our lab, we have had more than 60 mice in protocols with 24 weeks of HFD feeding, and these three have been the only deaths. It is also very rare for mice on the control diet to die. Other than the mice that died, all the other mice in the present study were generally healthy with the exception that the HFD mice were obese.

## Conclusion

Using CMR, GTTs, vascular reactivity, and histological studies, we showed that C57Bl/6 mice fed a HFD for 18–24 weeks are obese and have progressively increased LV mass, progressively decreased MPR, glucose intolerance, interstitial fibrosis, and reduced coronary arteriole reactivity with normal capillary density and no aortic plaque. These studies establish mice fed a HFD as a model system for the clinical scenario of obese and diabetic patients with reduced MPR in the absence of significant coronary artery disease. Future studies using CMR and gene-modified mice fed a HFD may shed light on key molecular mechanisms that underlie myocardial ischemia in obesity and diabetes-related cardiomyopathy.

## Abbreviations

AIF: Arterial input function
ANOVA: Analysis of variance
AUC: Area under the curve
BLOSM: Block LOw-rank Sparsity with Motion-guidance
CAD: Coronary artery disease
CS: Compressed sensing
CURE: Circumferential uniformity ratio estimate
DENSE: Displacement encoding with stimulated echoes
E_cc_: Global circumferential strain
E_cc-subendo_: Subendocardial circumferential strain
E_cc-subepi_: Subepicardial circumferential strain
EDV: End-diastolic volume
EF: Ejection fraction
ESV: End-systolic volume
FOV: Field of view
GTT: Glucose tolerance test
HFD: High-fat diet
LV: Left ventricular
LVEDWT: Left-ventricular wall thickness at end-diastole
LVESWT: Left-ventricular wall thickness at end-systole
LVWT: Left-ventricular wall thickness
MPR: Myocardial perfusion reserve
RAAS: Renin-angiotensin-aldosterone system
ROI: Region of interest
TE: Echo time
TF: Tissue function
TR: Repetition time
